# A Case of Ovariotomy

**Published:** 1878-06

**Authors:** William Meacher

**Affiliations:** Portage, Wisconsin


### A CASE OF OVARIOTOMY.

By William Meacher, M. D., Portage, Wisconsin.

The following case of ovariotomy, is published, not because it presents any novel features, but because in certain particulars it ought to be added to the literature already accumulated, in order to show the great importance of placing the patient under the best possible hygienic treatment.

In Nov., 1877, Miss F., aged 21, came to see me, by the advice of her physician—Dr. Blake, of Lodi, Wis. She had an ovarian tumor; but as she was not troubled in any way except by the abdominal enlargement, I advised letting it alone for the present.

On the 8th of January following, I tapped the cyst, removing a pailful of coffee-colored fluid, which was highly albuminous, and showed, under the microscope, crystals of cholesterine, and the "gorged granule" described by Peaslee. The gorged granule is, however, not considered so valuable a diagnostic sign as it was before Prof. I. N. Danforth, of Chicago, discovered it in the fluid of cystic disease of the kidney. She was tapped again in April. After this second tapping, it was evident that there was more than one cyst. Previous to this I had believed the tumor to be unilocular, the abdomen being uniformly distended, and uniformly diminished by the first tapping. Ovariotomy was performed by me during the forenoon of the 23d of June 1877, with the assistance of Drs. M. M. Davis, Wm. Fox, O. D. Coleman, Blake and McKeeby.

The patient's health had been rapidly failing for several weeks previous, but I was not made acquainted with the fact, or I should have operated sooner.

Squibb's chloroform was administered, the patient having previously taken $\frac{1}{4}$ gr. of morphia and 1 ℥ of whisky.

The incision was between five and six inches in length. Adhesions were found, very extensive and so strong that I was obliged to use both hands, and great force to tear them asunder.

The tumor proved to be multilocular in the full sense of the word: four different cysts had to be tapped before the former could be extracted through the incision. There were about fifteen different cysts, each one containing fluid differing in color and consistency from the others. Two, each about the size of a hen's egg, contained pus.

One vessel from the omentum was tied with a silk ligature, the ends cut short, and returned to the abdominal cavity. The pedicle was secured by a Spencer Wells'-clamp. The abdominal cavity was scrupulously sponged with fine, soft sponges, which were *rendered perfectly free from sand and dirt*, which I believe cannot always be said of the sponges used in such cases. The water used was well-water, filtered, and corbolized.

The wound was closed with silk sutures two-thirds of an inch apart, and was covered with a strip of cotton wadding, about three inches wide, and held in place by strips of adhesive plaster applied transversly, reaching entirely across the abdomen. A

piece of soft cotton cloth was laid over all, and a broad flannel bandage passed around the body completed the dressing.

The operation was a very difficult and tedious one, so much so that none of the physicians present thought the patient would survive. The operation was concluded at one p. m., and during the afternoon, as often as required, I gave small doses of morphine, and occasionally a tablespoonful of beef tea and a spoonful of wine. In the evening the pulse was 106; patient suffered but little, was very thirsty, took cold water freely. I left her at 9 o'clock, directing the nurse to give morphine when necessary to control pain, to give beef tea now and then, and to use the catheter every six hours.

Sunday morning, 24th. Found her pretty comfortable, pulse 106, though it had been up to 125 during the night; examined the stump and found a little bloody fluid oozing around it; applied solid perchloride of iron. She vomited some in the afternoon. A distressing cough, with more or less dyspnœa, came on in the afternoon. Patient said she used to be troubled with asthma before she had the tumor. In the evening the pulse was 120.

Monday, 25th, morning. The nurse informed me that the patient passed a bad night on account of bronchial trouble; pulse continued 120 all night. The cough and dyspnœa were not quite so bad during the day.

Tuesday morning, 26th. Was more comfortable, expectorating quite freely and breathing more easily; some soreness about the abdomen, but much less than could be expected from the cough and labored breathing; very little pain, skin moist. Pulse continued 110 during the day.

Wednesday morning, 27th. She looked bright and felt pretty well; she coughed a good deal the first part of the night but rested well the latter part; the pulse was 110. She took food well during the day; had very little pain or soreness; wound seemed to be well united. Should have removed the stitches but feared to do so on account of the cough. Urinated in the afternoon without the catheter.

Thursday morning, 28th. The pulse was 94; expectoration quite free; appetite improving; she wanted beefsteak and potatoes; no pain or soreness about the wound; removed the stitches.

Saturday, 30th. Found her doing well; bowels had acted the previous evening. Bronchial trouble had assumed an intermittent form. I therefore prescribed quinia 2 grs. every four hours.

Monday morning, July 2d. The cough was gone. The urine had become turbid and irritating. Prescribed carbonate of lithia in soda water.

Wednesday morning, 4th. Clamp came off; patient felt well; tongue clean; appetite good; no soreness about the abdomen; could lie on either side comfortably.

I did not see the patient again, but convalescence was rapid and complete.

Now this was certainly not a favorable case to begin with. The patient's health was rapidly failing, the tumor was one of the worst of its kind that I have had to deal with, the operation difficult and tedious, and the shock very severe. I believe the recovery in this case was, to say the least, largely due to the patient's surroundings. Her home is about 10 miles from Madison, situated in one of the most beautiful and healthy sections of this State. The house is a large, well built farm house. The patient's room was in the second story, a large airy room, with windows on two sides, provided with good shutters. The place was perfectly quiet, and the patient free from disturbance of any kind.

I believe that if every patient who had to undergo ovariotomy could be similarly situated, and at the same time could avail herself of the services of an ovariotomist of large experience, the percentage of deaths from this operation would be greatly reduced.

